# Onset of Postural Instability in Parkinson's Disease Depends on Age rather than Disease Duration

**DOI:** 10.1155/2022/6233835

**Published:** 2022-12-02

**Authors:** Denise Becker, Angelina Maric, Simon J. Schreiner, Fabian Büchele, Christian R. Baumann, Daniel Waldvogel

**Affiliations:** Department of Neurology, University Hospital Zurich, University of Zurich, Zurich 8091, Switzerland

## Abstract

**Background:**

Postural instability and falls are considered a major factor of impaired quality of life in patients with advanced Parkinson's disease (PD). The knowledge of the time at which postural instability occurs will help to provide the evidence required to introduce fall-prevention strategies at the right time in PD.

**Objective:**

To investigate whether postural instability of patients with different age at disease onset is associated with age or with disease duration of PD.

**Methods:**

Patients diagnosed with sporadic PD between 1991 and 2017 and postural instability (according to the International Parkinson and Movement Disorder Society Unified Parkinson's Disease Rating Scale (MDS-UPDRS) part III, item 3.12 postural instability) were included, with strict inclusion criteria including regular follow-ups, agreement on data use, and exclusion of comorbidities affecting the free stand.

**Results:**

Applying these strict inclusion criteria, we included 106 patients. Those younger than 50 years at PD onset took significantly longer to develop postural instability (*n* = 23 patients, median: 18.4 years) compared with patients with later onset of PD (50–70 years, *n* = 66, median: 14.2 years, *p* < 0.001; and >70 years, *n* = 17, median: 5.7 years, *p* < 0.001, Kruskal-Wallis test followed by Dunn's multiple comparisons test). There was no association between total MDS-UPDRS III (as a measure of motor symptom severity) at onset of postural instability.

**Conclusions:**

In PD, postural instability is primarily associated with the age of the patient and not with disease duration.

## 1. Introduction

Parkinson's disease (PD) is the second most common neurodegenerative disease in high-income countries. The incidence of PD is around 14 per 100,000 people overall and increases to around 160 per 100,000 for people older than 65 years (for review, see [1]). At the onset of PD, the three cardinal motor symptoms akinesia, rigor, and/or tremor are dominant. Later, axial symptoms become more prominent[1]. Axial symptoms include dysarthria, dysphagia, postural instability, and gait disorders, and those are usually less responsive to medication or surgical treatment (for review, see [3, 4]). Gait disorders in advanced PD are a major source of disability due to increased risk of falls, consecutive injuries, and hospitalizations [2] and reduced quality of life in PD [3, 4]. Of note, falls due to postural instability or balance problems within 3 years after disease onset are considered “red flags,” i.e., suggesting an atypical form of PD [1, 5].

The exact pathways needed to control and maintain postural stability are not known in detail, likely involving a complex interplay of networks ranging from the spinal cord through the brainstem and cerebellum to frontal and basal ganglia circuits. Maintaining postural stability is an extraordinarily complex task [3], and it is therefore not surprising that cognitive impairment correlates with postural instability and falls [6, 7].

Early studies stated that postural instability occurs 5–7 years after disease onset [8]. However, more recent studies report postural instability occurring 10–15 years after first diagnosis [9–11]. Despite the tremendous impact of postural instability on independence and quality of life, its time course remains debated. There are hints that a clinical subscore (combining several axial items retrieved from the MDS-UPDRS including postural instability) or the clinical score “Höhn and Yahr” is more correlated with age in patients with short (on average <10 years) disease duration [12–14]. But so far, this has not been investigated.

We were interested in determining how duration of disease and age correlate with the occurrence of postural instability. It has recently been shown that cognitive decline correlates more with age than with disease duration [15, 16] and we aimed to investigate whether the same might apply for postural instability.

## 2. Patients and Methods

### 2.1. Ethical Compliance Statement

This study was conducted in accordance with the Declaration of Helsinki and after ethical approval of local authorities (Kantonale Ethikkommission Zürich, BASEC No. 2021-00170).

### 2.2. Patients

We retrospectively included patients diagnosed with sporadic PD between 1991 and 2017 from our movement disorders outpatient clinic. Inclusion criteria were diagnosis of sporadic PD according to international consensus criteria [1], occurrence of postural instability, and regular follow-up including standard clinical examinations. Routine clinical assessments in our outpatient clinic included the International Parkinson and Movement Disorder Society Unified Parkinson's Disease Rating Scale (MDS-UPDRS III), part III (or in earlier consultations the Unified Parkinson's Disease Rating Scale UPDRS), motor phenotype [17], treatment (deep brain stimulation and medication), and sporadic assessment of cognitive functions (Montréal Cognitive Assessment (MoCA)). Motor examinations were obtained on habitual medical treatment, which challenges a reliable estimation of motor progression. We therefore focused on axial symptoms, which are less responsive to L-dopa and inevitably arise despite treatment, allowing for an accurate assessment under habitual medical treatment. It has been reported that postural instability is the best marker for disease progression on dopaminergic therapy [18].

To measure postural instability, we used the subscore item of the “pull test” in the MDS-UPDRS. The pull test is the clinical gold standard examination to assess postural instability and is therefore widely used [19, 20]. It is easy to perform and correlates with risk of falls [21]. However, regarding sensitivity and specificity, the pull test is worse than other tests that require technical equipment or a combination of several tests. We used the MDS-UPDRS III subscore of the pull test (item 3.12) or the equivalent UPDRS III subscore, both ranging from 0 to 4 points, as a measure of postural instability. To exclude any ambiguity and rater bias for the purpose of this study, we defined patients with postural instability as patients who rated ≥2 points in UPDRS part III (=no postural reaction after pull test) or ≥3 points in MDS-UPDRS part III (=no postural reaction after pull test) on two consecutive visits. This corresponds to a distinct impairment of postural stability, which could be reproducibly assessed.

Primary outcome parameters were age and disease duration at onset of postural instability. Disease duration was the time from onset of PD until the patient developed postural instability. Onset of PD was retrospectively assessed by the clinician during routine visits in the outpatient clinic according to the criteria for prodromal PD by the International Parkinson and Movement Disorder Society [22].

### 2.3. Quantification and Statistics

Statistical comparisons of groups were done using the Mann–Whitney test (for comparing two groups) and with Kruskal–Wallis test followed by Dunn's multiple comparisons test (for comparing three or more groups). Spearman correlation coefficients were calculated to assess associations between outcome variables. All statistical analysis was done with GraphPad Prism 6 (GraphPad software, USA). *p* values of less than 0.05 were considered significant.

### 2.4. Digital Illustrations

Figures were prepared using Inkscape (https://www.inkscape.org).

## 3. Results

### 3.1. Patient Characteristics

**Figure 1:**
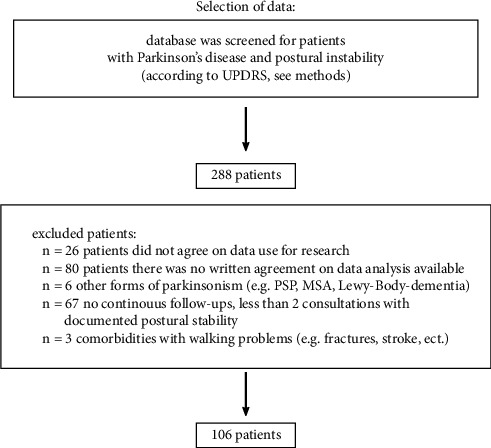
Criteria for patient selection. Patient database was screened for patients with Parkinson's disease who developed postural stability. 288 patients were found. After excluding the patients according to the exclusion criteria, 106 patients were left. UPDRS: Unified Parkinson's Disease Rating Scale, PSP: progressive supranuclear palsy, and MSA: multiple system atrophy.

We identified 288 PD patients who suffered from severe postural instability (MDS-UPDRS III score, item 3.12. of at least 3 points, or UPDRS III of at least 2 points, see methods part). Out of these, 182 patients were excluded: 26 patients did not agree on use of their data for research, for 80 patients, no statement about data use was available (no address and patient died already), 67 patients were excluded because of missing long-term follow-ups, for 6 patients, the initial diagnosis was changed to other forms of Parkinsonism, and 3 patients had comorbidities (stroke and fracture) that made it impossible to measure postural stability (see Figure 1).

Demographics and clinical characteristics of patients with postural instability are shown in Table 1.

### 3.2. Onset of Postural Instability Occurs Later in Disease Course in Patients with Early Manifestation of PD

**Figure 2:**
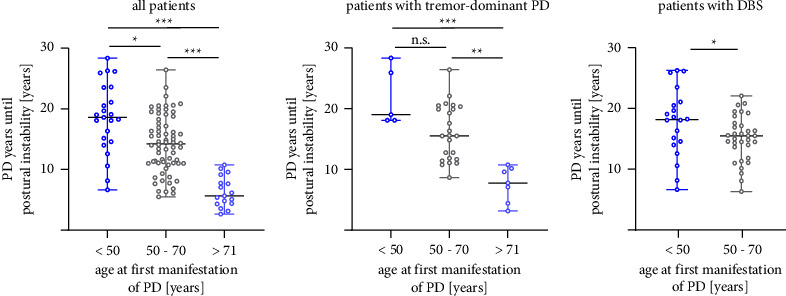
Onset of postural instability occurs later in the disease course in patients with early manifestation of PD. (a) Patients with onset of PD < 50 years (*n* = 23 patients, left) have on average 18.6 (median; range: 6.7–28.3) PD years before the onset of postural instability. This time is significantly different compared to patients with PD onset at 50–70 years (middle, *n* = 66 patients, median: 14.2 years, range: 5.5–26.4) and >70 years (right bar, *n* = 17, median: 5.6 years, range: 2.7–10.8 years); (b) From the graph in (a), only the tremor-dominant patients were selected. Patients with onset of PD < 50 years (*n* = 5 patients, left) have median 19 (range: 18.8–28.33) PD years before onset of postural instability. This is not different compared with patients with first manifestation between 50 and 70 years (middle, *n* = 24 patients, median: 15.5 years, range: 8.67–26.42) but both groups are significant different to manifestation of PD > 70 years (right bar, *n* = 7, median: 7.8 years, range: 3.2–10.8 years); (c) From the graph in (a), only the patients treated with DBS were selected. Patients with manifestation of PD < 50 years (*n* = 20 patients, left) have median 18.2 (range: 6.3–22.1) PD years before onset of postural instability. This is significantly longer compared with patients with first manifestation between 50 and 70 years (right, *n* = 33 patients, median: 15.5 years, range: 6.33–22.1). In (a) and (b), we used Kruskal-Wallis test followed by Dunn's multiple comparisons test, in (c) Mann-Whitney test; the plots show median with range and single values ^*∗*^*p* value <0.05, ^*∗∗*^*p* < 0.01, ^*∗∗∗*^*p* < 0.001.

In the final sample (*n* = 106), we stratified the patients into three groups according to their age of onset of PD (<50 years, 50–70 years, and >71 years), and data were analyzed for the first occurrence of postural instability (Figure 2(a)). Here, we saw that patients with onset under 50 years took significantly longer to develop postural instability compared to patients with onset at 51–70 and >71 years. This suggests that the age of PD onset is very important for the time when postural instability occurs.

At onset of postural instability, the groups had comparable total scores in the MDS-UPDRS III (<50 years: *n* = 23, median 28, range 14–64; 50–70 years: *n* = 66, median = 31.5, range 14–66 points, and >71 years: *n* = 17, median 30, range 21–76 points, all comparisons n.s., Kruskal-Wallis test followed by Dunn's multiple comparisons test; data not shown). In this patient cohort, 36 patients suffered from tremor-dominant PD. A separate analysis of patients with tremor-dominant PD revealed a significant difference in time to the onset of postural instability in the three groups with different age at onset of PD (<50 years, 50–70 years, and >71 years) (Figure 2(b)).

Notably, 20 patients (of 23, target region: substantia nigra, STN) in the group <50 years and 33 patients (out of 66, target region: 31x STN, 1x globus pallidus internus, and 1x ventralis intermediate nucleus of the thalamus) in the group 50–70 years were treated with deep brain stimulation (DBS). When the data with DBS treated patients were analyzed, the effect was still significant (Figure 2(c)).

The median time to develop postural instability after implantation of DBS was 3.33 years (range: 0.08–8.83 years) in both groups. In the group with first manifestation of PD over 71 years of age, no one was treated with deep brain stimulation.

At onset of postural instability, cognitive abilities were not significantly different between the groups. The groups had comparable results in the MoCA test (<50 years, *n* = 21, median 24, range 4–29; 50–70 years, *n* = 51, median = 21.5, range 10–29 points, and >71 years *n* = 12, median 23.5, range 16–28 points, all comparisons n.s., Kruskal-Wallis test followed by Dunn's multiple comparisons test; data not shown). In PD, usually a score of under 26 points is considered a mild cognitive impairment. Thus, all groups were on average showing cognitive impairment [23]. However, the data were very variable, ranging from 4 to 29 points on the MoCA test.

### 3.3. Postural Instability Correlates with the Age of the Patient and Not Primarily with Disease Duration

**Figure 3:**
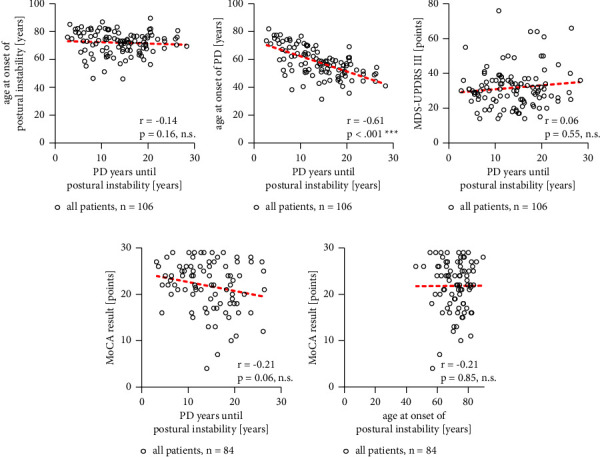
Age at onset of postural instability does not correlate with disease duration.  Figure 3 shows the distribution of (a) age at onset of postural instability, (b) age at onset of PD, (c) total MDS-UPDRS III scores, MoCA test results in correlation to the disease duration of PD (graph (d)) (*x*-Axis: PD years until postural stability) and MoCA test results in correlation to age at onset of postural instability (e). The *n*-numbers are indicated above the graphs, The red dotted line shows the calculated linear regression. Correlation analysis: *R* values and *p* values are indicated in the graphs in the right corner. ^*∗*^Spearman's test, ^*∗*^*p* < 0.05 statistically significant.

Figure 3(a) shows on the *y*-axis the age of the patient at which postural instability was diagnosed. On the *x*-axis, the interval between disease onset and postural instability onset is depicted. This correlation is not significant (see Figure 3(a), correlation coefficient: −0.14, *p* = 0.16, Spearman's test). This means the age of the patient primarily defines the onset of postural instability and not disease duration. This is confirmed by the finding that there was a significant association between the age at first manifestation of PD with the age at onset of postural instability (see Figure 3(b), correlation coefficient: −0.61, *p* < 0.001, Spearman's test), i.e., the older the patient at disease onset is, the sooner the patient develops postural instability.

The results of the MoCA test and total MDS-UPDRS III scores did not correlate with the disease duration until onset of postural instability (see Figures 3(c) and 3(d)). The results of the MoCA test did not correlate with the age at onset of postural instability (Figure 3(e)).

### 3.4. Age at Onset of Postural Instability in the Majority of PD Patients Is >60 Years

In our study, the average age was 72.06 years (median: 73.29 years; range: 46.08–89.67 years) at the onset of postural instability. As shown in Figure 3(b), the vast majority (95.5%) of patients developed postural instability over 60 years of age and only three patients developed it under the age of 55 years, at the age of 46.1, 46.4, and 50, respectively.

## 4. Discussion

Postural instability is one of the most disabling features of late stage PD and is very difficult to treat. Our retrospective analysis of 106 patients over a time course of up to 28 years after onset of PD provides new insights into the onset of postural instability during disease progression. Our data show that the onset of postural instability is mainly driven by age and not disease duration, i.e., patients with early manifestation of PD take significantly longer to develop postural instability.

It has been reported in earlier studies that older age is a risk factor for more rapid progression of PD [24, 25]. A retrospective analysis showed a high hazard rate for falls and postural instability with patients diagnosed after the age of 70 years [14]. Our data have the strength that we have long follow-ups (up to 28 years) and that we have in terms of age a very diverse patient population (onset of postural instability in patients 46–89 years old).

We can show a clear correlation of age of onset of PD with onset of postural instability. A subgroup analysis reveals that this correlation is also true for patients suffering from tremor-dominant PD. This is interesting as tremor-dominant PD is usually known to have a more moderate progression [26], even if in early disease the differences are not so pronounced [27]. However, recently there are efforts to find better characterizations of the motor subtypes via computational modeling and it is possible that motor phenotypes will be characterized differently in the future [26, 28]. Age at onset of PD also drives the onset of postural instability when only patients treated with deep brain stimulation are analyzed. This is as expected as deep brain stimulation has a weak effect on postural instability [29].

Postural stability like cognition depends on the integrity of widespread networks [6, 30, 31]. It is therefore not surprising that most patients with severe postural instability are also cognitively impaired. On average we see at onset of postural instability a reduced score in the MoCA test (median: 22 points, see Table 1) which is highly suggestive of mild cognitive impairment in PD [23]. Concerning cognition, previous studies report similar findings for postural instability, i.e., patients with younger onset of PD take longer to develop cognitive deficits during disease progression compared with late-onset PD patients [32]. Recent work highlights also the significant contribution of cholinergic disturbance to gait [7, 33, 34]. It has been shown that PD patients with mild cognitive impairment benefit from treatment with an acetylcholine-esterase inhibitor resulting in less falls and gait disturbances [35].

Of course, this study bears the limitations that apply to a purely retrospective study, particularly that our cohort of patients consists of patients from a university hospital outpatient clinic, where many of the more severely affected patients are referred. However, our data were in line with other studies, i.e., the Sydney cohort [10] reported in 2005 an age of 67.5 years for onset of falls, whereas a study 5 years later [36] reported a higher age of 70.4 years. In our cohort, the average age was 72.1 ± 8.4 years for the onset of severe postural instability. This slightly higher age might be due to improved treatment in the past years.

The diagnostic criteria for PD state that early onset of postural instability within the first three years after diagnosis of PD is a “red flag” and very likely indicates an atypical Parkinson syndrome [1, 5]. This is in line with our data. Of particular clinical importance in this context is also our observation that a minority of patients developed postural instability before the age of 60 years. We therefore would like to suggest that severe postural instability before the age of 60 years should be considered a “red flag” when diagnosing PD.

## Figures and Tables

**Table 1:** Summary of demographics.

Variable	Median	Range
*N* = 106 patients; males/females: 58/48		
Age at first manifestation of PD (years)	57	31.4–82.1
Age at onset of postural instability (years)	73.29	46.1–89.7
Höhn and Yahr scale (*n* = 83 patients)	3	2–3
MoCA test (points) (*n* = 84 patients)	22	4–29
Time between conduction of MoCA test and initial manifestation of postural instability (years)	1.25	0–8
Treated with DBS when postural instability occurred (%)	56.2	
Total MDS-UPDRS III at postural instability onset (points)	30	14–76

Table 1 shows a summary of the demographics and clinical characteristics of the patients investigated in this study. DBS: deep brain stimulation; PD: Parkinson's disease; MoCA: Montréal Cognitive Assessment; MDS-UPDRS: International Parkinson and Movement Disorder Society Unified Parkinson's Disease Rating Scale.

## Data Availability

The data presented in this study are available on request from the corresponding author.
